# Influence of Action-Effect Associations Acquired by Ideomotor Learning on Imitation

**DOI:** 10.1371/journal.pone.0121617

**Published:** 2015-03-20

**Authors:** Frédérique Bunlon, Peter J. Marshall, Lorna C. Quandt, Cedric A. Bouquet

**Affiliations:** 1 University of Poitiers, CNRS, Poitiers, France; 2 Temple University, Philadelphia, Pennsylvania, United States of America; 3 University of Pennsylvania, Philadelphia, Pennsylvania, United States of America; UCLA, UNITED STATES

## Abstract

According to the ideomotor theory, actions are represented in terms of their perceptual effects, offering a solution for the correspondence problem of imitation (how to translate the observed action into a corresponding motor output). This effect-based coding of action is assumed to be acquired through action-effect learning. Accordingly, performing an action leads to the integration of the perceptual codes of the action effects with the motor commands that brought them about. While ideomotor theory is invoked to account for imitation, the influence of action-effect learning on imitative behavior remains unexplored. In two experiments, imitative performance was measured in a reaction time task following a phase of action-effect acquisition. During action-effect acquisition, participants freely executed a finger movement (index or little finger lifting), and then observed a similar (compatible learning) or a different (incompatible learning) movement. In Experiment 1, finger movements of left and right hands were presented as action-effects during acquisition. In Experiment 2, only right-hand finger movements were presented during action-effect acquisition and in the imitation task the observed hands were oriented orthogonally to participants’ hands in order to avoid spatial congruency effects. Experiments 1 and 2 showed that imitative performance was improved after compatible learning, compared to incompatible learning. In Experiment 2, although action-effect learning involved perception of finger movements of right hand only, imitative capabilities of right- and left-hand finger movements were equally affected. These results indicate that an observed movement stimulus processed as the effect of an action can later prime execution of that action, confirming the ideomotor approach to imitation. We further discuss these findings in relation to previous studies of action-effect learning and in the framework of current ideomotor approaches to imitation.

## INTRODUCTION

Imitation refers to the overt reproduction of an observed action. In order to imitate, the observer is required to translate perceived aspects of another individual’s behaviour into motor commands [1–2]. A central question in the imitation literature, known as the correspondence problem, is how sensory codes are transformed into motor codes [2–3].

A solution to the correspondence problem can be provided by assuming a common coding between perception and action [2, 4–6]. For example, the active intermodal mapping hypothesis [7] explains infant facial imitation by postulating a comparison process between the observer’s proprioceptive feedback and visual information about the observed act. Such an intermodal mapping is possible because perceived and executed actions are coded within a common representational framework in terms of the relations between organs, that is, spatial arrangements between significant body parts (e.g. tongue and lips) [7]. The idea that action and perception share a common representational format is also a central claim of ideomotor theories ([2, 8]; for a review, see [9]). Within the ideomotor framework, it is assumed that actions are controlled by representations of their perceivable effects, including body-related information (proprioceptive feedback and visual information about the position of the arm during and/or after a movement) and more distal, remote action-effects (changes in the environment) [8–10]. This approach is based on the core assumption that repeatedly performing a movement and perceiving its effects in close temporal succession results in bi-directional associations between the action’s motor codes and the codes of sensory action effects (i.e. *ideomotor learning* or *action-effect binding*) [2, 11–12]. These associations form the basis of motor representations and once they are established, it is assumed that anticipating or thinking of the perceptual consequences of an action elicits execution of that action. Importantly, this principle extends to perception: an action can be triggered by a perceptual event which is similar to the effects associated with this action—such as when perceiving another’s action [2]. The ideomotor theory can thus account for imitation: when seeing somebody else’s action and its consequences, the action plans that would lead to those consequences are activated in the observer [13].

A number of convergent lines of research support the common coding between perceived and executed actions suggested by the ideomotor theory. At a neurophysiological level, the discovery of *mirror neurons* in area F5 of the monkey premotor cortex provides evidence that the observation and execution of action share a common neural representation [14]. Mirror neurons fire both when the monkey performs an action and when the monkey observes the same action performed by the experimenter. A similar *mirror matching system* seems to exist in humans, although the nature and function of this system has been debated. Neurophysiological studies (e.g., EEG, fMRI) have revealed a circuit—including the posterior part of the superior temporal sulcus, the rostral part of the inferior parietal lobule, the posterior part of the inferior frontal gyrus (IFG) and adjacent ventral premotor cortex—which is activated when people observe someone else executing an action, and also when they are themselves doing the same action (for reviews see [6, 13]).

At a behavioural level, the ideomotor theory predicts that action execution or initiation is facilitated by the observation of a similar action, whereas observing a different action interferes with action execution. In line with this prediction, it has been demonstrated that observation of finger tapping movement interfered with execution of a finger lifting movement and vice versa [15]. Similar motor interference vs. facilitation effects have been demonstrated in numerous kinds of action, such as hand opening and closing [16], mouth movement [17] and arm movement [18].

As seen above, another main assumption of the ideomotor theory is that the association between perceptual effects and motor codes is built through experience: we learn to associate our action with their effects. This assumption has been corroborated by several studies showing that perception of a learned action effect elicits the response that had previously caused this effect [12, 19–23]. For example, in a recent experiment [19], participants underwent an acquisition phase, in which a self-produced key-press (left/right) triggered a specific tone (low pitch/high pitch). In a later test phase, the same tones were used as imperative stimuli. Half of the participants (the acquisition-compatible subgroup) had to respond with the key that preceded the tone in the acquisition phase. The other half (the acquisition-incompatible subgroup) had to respond with the key that preceded the other tone in the acquisition phase. The authors observed that keypresses were produced faster when responding to a tone that the action had previously triggered (acquisition-compatible) than when responding to a tone that had triggered the alternative tone (acquisition-incompatible) [19]. This result is interpreted as evidence for ideomotor (action-effect) learning: the perception of a learned action effect activates the action it is associated with.

Associating remote, environmental effects with an action may play an important role in imitation [24–26]. Most action goals refer to remote effects and/or target objects (turning a light on, reaching for a cup, etc.) [10] and studies in infants have demonstrated the importance of goals for imitation [24, 26–27]: they tend to reproduce the effects of the action in the environment without necessarily using the same means (movements) as the model [26, 28]. When imitating goal-directed actions, the perception of the action’s consequences in the environment activates the action which is most strongly associated with this remote effect, leading to imitation of the goal, without necessarily copying the exact movements of the model [24].

However, we are also able to imitate meaningless movements or non-goal-directed, intransitive actions (e.g. communicative gestures, dance movements). When imitating this type of action, the movement itself is the goal and we aim to replicate the motor part of the action, [24]. Here it is assumed that the perception of body movements triggers the corresponding motor commands, enabling the exact movements of the model to be copied [13, 29–30]. This mapping of observed movements onto corresponding motor commands in the observer is referred to as *motor resonance* [13, 31]. Some current theories consider that copying movements is the core of the imitation process [29] and according to recent models, motor resonance is crucial for imitative learning [25, 31].

The mechanisms of action control proposed by the ideomotor theory also provide a framework for imitation. Within this framework, an observed action can be copied by the observer because the cognitive codes representing the observed movement or its remote effects are associated with the motor codes of the action producing these effects. This association results from prior sensorimotor experiences linking execution of action with its perceptual effects (action-effect learning). However, for a more complete account of imitative behaviour by ideomotor principles, it is necessary to demonstrate that action-effect learning can influence our capability to imitate specific body movements as the theory predicts.

The capacity to resonate with and copy the specific movements of a model requires acquired associations between the visual percept of the movements and their motor codes [13, 25, 32]. This type of action-effect learning and its potential influence on imitation have been largely ignored in the ideomotor learning literature. On the one hand, there is little experimental evidence of ideomotor learning where the learned action effects consist of movement stimuli. Previous research on action-effect learning typically investigated integration of new remote action-effects in the environment, consisting of inanimate stimuli (e.g., pitch tones, visual letters, words, symbols; [12, 19–23; 33–35]; but see [36] for use of social stimuli). On the other hand, action-effect learning was assessed by presenting these former effect stimuli as imperative stimuli in a choice response task—i.e. no imitation was involved.

Thus, an important, but unexplored, question is whether imitative capabilities can be modified by the acquisition of body-related action-effects through ideomotor learning. Investigating this question is crucial to confirm the ideomotor account of imitation and may contribute to better understanding of the underlying mirror matching processes. One possible approach is to demonstrate that an observed movement processed as the effect of an action can acquire the capacity to prime that action in subsequent imitation.

One recent study investigated the influence of ideomotor learning on imitative behaviour [37]. In this study, the authors found that after a learning phase where hand movement triggered observation of foot movement, the body priming effect induced by observation of hand movements was reduced. Such a body priming effect is an expression of automatic imitation, which refers to an unintentional and automatic tendency to reproduce an observed task-irrelevant movement [38]. In contrast, intentional imitation refers to a voluntary overt reproduction of another’s action or movement.

It is necessary to extend the examination of the influence of ideomotor learning to intentional imitation for several reasons. First, as explained above, the demonstration of the influence of ideomotor learning on imitation is limited, and it is thus important to replicate and extend previous findings. Second, evidence from behavioural and neuroimaging studies support the view that motor resonance processes are at the core of both intentional and automatic imitation [1, 13, 39–40]. Nonetheless, observing an action with the intention to imitate is known to involve different brain processes than those involved when passively observing an action [41]. Besides the involvement of the fronto-parietal mirror matching system, prefrontal areas may also be involved in intentional imitation, which might reflect the maintenance of motor representations, or top down control of action representations [13, 42]. Finally, it is particularly relevant to test the prediction of ideomotor theory on intentional imitation, since the ideomotor approach emphasizes the importance of intentional processes in guiding imitative behaviours [2, 9].

The aim of the present study was to test whether intentional imitation performance is affected by prior action-effect learning where execution of a self-selected action was followed by perception of a same vs. different action. Our rationale was the following: under real life conditions, the execution of a movement A (e.g., a hand movement) is often accompanied with perceptual effects, including seeing movement A. The repetitive experience of executing A leads to the association of the visual codes representing A with the motor codes for executing A, as predicted by the ideomotor theory. We can then imitate movement A because seeing A triggers the corresponding motor codes. Building on this logic, we hypothesized that a learning condition where seeing movement A is the ‘new’ effect of an action B should lead to the integration of the visual percept of A with the motor codes for B. Hence, this learned action-effect should then interfere with the capability to imitate movement A, since seeing A now triggers the motor codes associated with the execution of B. We predicted that imitative performance following this type of learning should be altered compared to following a learning condition in which seeing A is the consequence of executing A.

## EXPERIMENT 1

In this experiment, imitation was measured in a reaction time (RT) task where participants were instructed to imitate finger movements displayed on a screen. The task therefore involved intentional imitation, in that participants voluntarily reproduced a target movement. We tested whether imitative performance in the RT task was influenced by prior response-effect learning. We contrasted two types of action-effect mapping. The Compatible mapping (CM) group underwent an acquisition phase in which execution of a self-selected action A (e.g. index finger lifting) was followed by observation of the same action. The Incompatible mapping (IM) group underwent an acquisition phase in which execution of an action A (e.g. index finger lifting) was followed by observation of an action B (little finger lifting).

In addition to our main predication that IM learning should impair subsequent imitative performance compared to CM learning, specific predictions regarding performance in the acquisition phase could be made. Within the ideomotor framework, representations of the response effects are automatically activated in the course of initiating the response. In line with this, previous studies have demonstrated that under conditions where participants produce responses that consistently lead to spatially incompatible effects, RTs are longer compared to a condition where the anticipated effects are compatible with the executed response [43–45]. Furthermore, the influence of anticipated effects has been found to increase the later the response is executed [43–44], suggesting that the activation of the effect codes is time consuming. In the acquisition phase, we thus expected longer RTs in the IM group compared to the CM group and that this difference should increase with RT. In the acquisition phase, we thus expected longer RTs in the IM group compared to the CM group and that this difference should increase with RT.

### Method

#### Participants

Forty-six undergraduates from the University of Poitiers took part in Experiment 1, in exchange for course credit. The participants (7 males) ranged in age from 18 to 33 years (mean age = 20.0 years). All were right handed, had normal or corrected-to-normal vision and were naïve with respect to the purpose of the experiment. Participants were randomly assigned to CM and IM groups.

Each participant read and signed an informed consent form prior to taking part in the experiment. All aspects of this study were performed in accordance with the ethical standards set out in the 1964 Declaration of Helsinki. The study was approved by the local ethic committee of the laboratory—Center for Research in Cognition and Learning (CeRCA)—and was conducted in accordance with national norms and guidelines for the protection of human subjects.

#### Apparatus and Material

The presentation of stimuli and the registration of manual responses were controlled by E-prime software (version 2.0, http://www.pstnet.com/). Stimuli were presented on a 20-in. Nokia monitor.

Participants were seated approximately 60 cm from the screen, with their index and little fingers of the right hand resting on two buttons on a response box placed in front of them. The distance between these two buttons was approximately 9 cm, so that when a participant rested his/her hand on the box, index and little fingers were pressing the left and right buttons, respectively.

In the different tasks, participants observed apparent movements of a finger (on a hand) displayed on the screen. The sequences of stimuli comprised 2 pictures of a female right or left hand. The hand had no distinguishing features and could be considered as a neutral hand. It was presented in color on a black background in the middle of the screen, in the same axis as the participant’s hand, as if viewed from above. The hand occupied approximately 7.6° of visual angle horizontally and 13.3° vertically.

**Fig 1:**
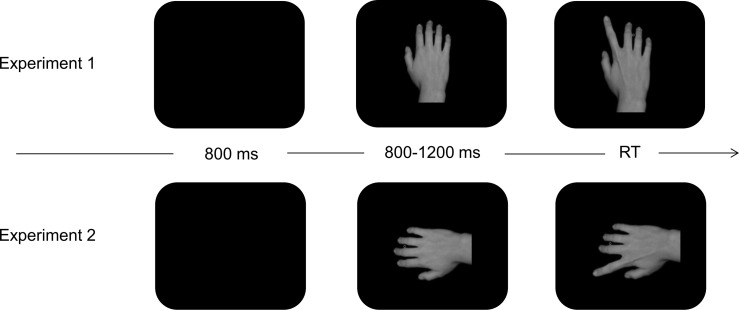
Examples of the stimuli used for the intentional imitation task in Experiment 1 (top panel) and Experiment 2 (lower panel). Each trial started with a blank interval of 800 ms. Then a picture of the hand in a neutral position was presented for 800 or 1200 ms, followed by the imperative stimulus: a picture of the hand with a lifted index or little finger (displayed until participant's response). Participants had to respond by lifting the same finger.

Apparent motion of the fingers was produced by presenting a picture of the hand in a resting (neutral) position followed by a picture of the same hand with the index or little finger lifted and slightly abducted (Fig. 1). The replacement of the initial image by the final finger position produced apparent motion. The finger movements subtended an angle of 2.6° (index) and 2.2° (little) from the neutral position. The left hand stimuli were made by reflecting the right hand pictures along the y-axis.

These stimuli were used as target stimuli (i.e., the movements to imitate) in the intentional imitation task and as sensory feedback in the acquisition phase.

#### Intentional imitation task

Participants from both groups were asked to use their right hand to imitate the movements of the index or little finger of a left or right hand. When the index finger was lifted on the screen, they had to lift their index finger, and when the little finger was lifted, they had to lift their little finger. Participants were instructed to respond as fast as possible while avoiding mistakes.

Each trial began with a blank interval of 800 ms, followed by the picture of the right or left hand in the neutral position, presented for 800 or 1200 ms (randomly selected). This picture was then replaced by the imperative stimulus: a picture of the same hand with a displacement of index or little finger (see above). This stimulus remained on the screen until participant’s response. The next trial started immediately after the response, unless an error was made, in which case an error message was displayed for 750 ms.

Each block consisted of 48 trials, with hand (left or right) and movement (little or index finger) being selected randomly so that each hand × movement combination was presented 12 times.

The participants performed 5 blocks before and 5 blocks immediately after the acquisition phase (see below). Participants were allowed to take a break and remove their hand from the response box between each block. After each block, participants received feedback on their performance (mean RT and accuracy).

Before the experimental trials, participants completed 2 blocks of 8 trials, in order to ensure that they understood the task, followed by one practice block of 48 trials. Practice trials were excluded from further analysis.

#### Acquisition phase

This acquisition phase took place immediately after a pre-test baseline. During this phase, participants from both groups received the same instruction: when a white dot appeared on the screen, they had to lift either the index or little finger.

Each trial started with the presentation of a right or left hand in a neutral position (as in the procedure described above for pre and post-test). After a delay of 800–1200 ms, a white dot appeared approximately in the center of the screen, between the index and middle fingers. The participants were instructed to respond by lifting either the index or the little finger. They were asked to try to lift the index and little finger randomly. The participant’s response was immediately followed by the display of one picture of the hand with the index or the little finger lifted. This final picture was displayed for 550 ms. A blank screen was then presented for 800 ms before the next trial began

**Fig 2:**
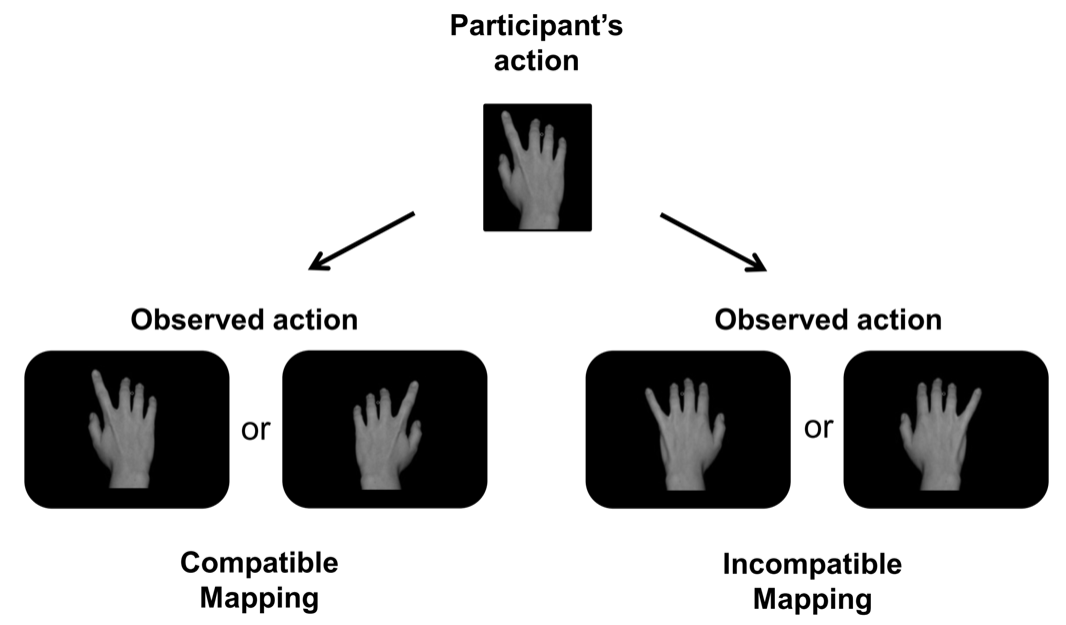
Illustration of the acquisition phase. The participant’s movement was followed by the presentation of similar (Compatible Mapping) or different (Incompatible Mapping) movement. In experiment 2, only right hands were presented as visual feedback.

On each trial, the participant’s movement was thus followed by an apparent movement of the finger on the screen. For the CM group, when the participant lifted the index finger (or little finger), this triggered lifting of the index finger (or little finger) on the screen. For the IM group, when the participant lifted the index finger, this triggered lifting of the little finger on the screen, and vice versa (Fig. 2).

Participants completed 10 blocks of 40 trials. They were allowed to take a break between each block and a 2-min pause was imposed between the 5^th^ and the 10^th^ block. Participants were not told this part of the experiment was a “learning” phase.

To ensure that participants remained focused on the screen, in 8.25% of the trials a digit (between 1 and 9) appeared on the tip of the index or little finger of the hand displayed. Participants were asked to tell the experimenter which digit appeared each time they saw one.

In addition, a cardboard cover was placed above the hand of the participants so that they could not see their own fingers moving. We predicted this procedure would ensure that the visual perception of the participant’s own movement would not affect the integration of the perceptual features of the action displayed on the screen with motor commands.

Importantly, during this acquisition phase, participants were instructed to lift their index or little finger at random and to avoid using a strategy—such as alternating systematically between responses or alternating between series of the same finger movement. Respecting this instruction was important because it promoted the selection of a given movement on every trial. Thus, this favored temporal contiguity between selection of action and visual feedback. Because temporal contiguity is crucial for action—effect learning [46], we assumed this procedure was necessary to create a link between the selected action and its visual effect. Participants’ responses were checked offline to determine if they indeed followed the rule, i.e. they produced approximately the same number of response alternations and repetitions. We also tested if the CM and IM groups were similar in this respect.

### Results and discussion

Six participants in Experiment 1 were excluded from all further analyses. Five were excluded because they exhibited atypical patterns during the acquisition phase, with more than 75% of responses following an alternating pattern. One participant was excluded because his mean RT in the imitation task was more than three standard deviations from the group mean. This exclusion affects none of the results described below, other than increasing the statistical power to detect an effect of acquisition condition.

#### Acquisition

The participants produced, on average, 63% alternating responses (i.e., the proportions of trials in which the finger movement on a given trial was different from that on the previous trial). The repetition/alternation ratio was similar in CM and IM groups, Pearson's chi-square = 0.83, *p* = .36.

The mean RTs of the participants in the CM and IM groups were 404 ms (SD = 64) and 462 ms (SD = 128), respectively. The difference between groups did not reach significance, *F*(1, 38) = 3.33, *p* = .08, η²_p_ = .081. In order to examine the temporal dynamics of the between-group difference, we conducted a distribution analysis on the RT data, using the Vincentization method [47]. RTs were rank ordered separately for each participant, divided into five bins (quintiles), and the mean RT for each bin and each participant was then calculated.

As expected, the difference between IM and CM groups tended to numerically increase with RT (IM mean RT—CM mean RT = 34 ms at bin 1, 45 ms at bin 2, 57 ms at bin 3, 67 ms at bin 4 and 98 ms at bin 5). An ANOVA, with Action-effect mapping (CM vs. IM) as between-participants factor and Bins (1–5) as within-participant factor, revealed a significant effect of Bins, *F*(4, 152) = 246, *p* <. 001, η ²_p_ = .86, and a marginally significant effect of Action-effect mapping, *F*(1, 38) = 3.87, *p* = .057, η ²_p_ = .092, but the interaction between these two factors was not significant, *F*(4, 152) = 1.39, *p* = .24, η ²_p_ = .035.

#### Intentional imitation task

For the RT analysis, we discarded all trials with an error (4.5%). For the remaining trials with correct responses, trials with RT below 100 ms or above 2.5 standard deviations of the mean of each participant were excluded (2.5% of the otherwise valid RTs).

We first tested for potential differences in performance between the CM and IM groups before acquisition. An ANOVA conducted on RTs with Action-effect mapping (CM vs. IM) as a between-subjects factor indicated no significant difference between groups, F(1, 38) = 0.62, *p* = .43, η²_p_ = .016. The same analysis conducted on error rates indicated a significant effect, F(1, 38) = 4.50, *p* = .04, η²_p_ = .10, due to more errors in the CM (5.7%) than the IM group (4.2%).

**Fig 3:**
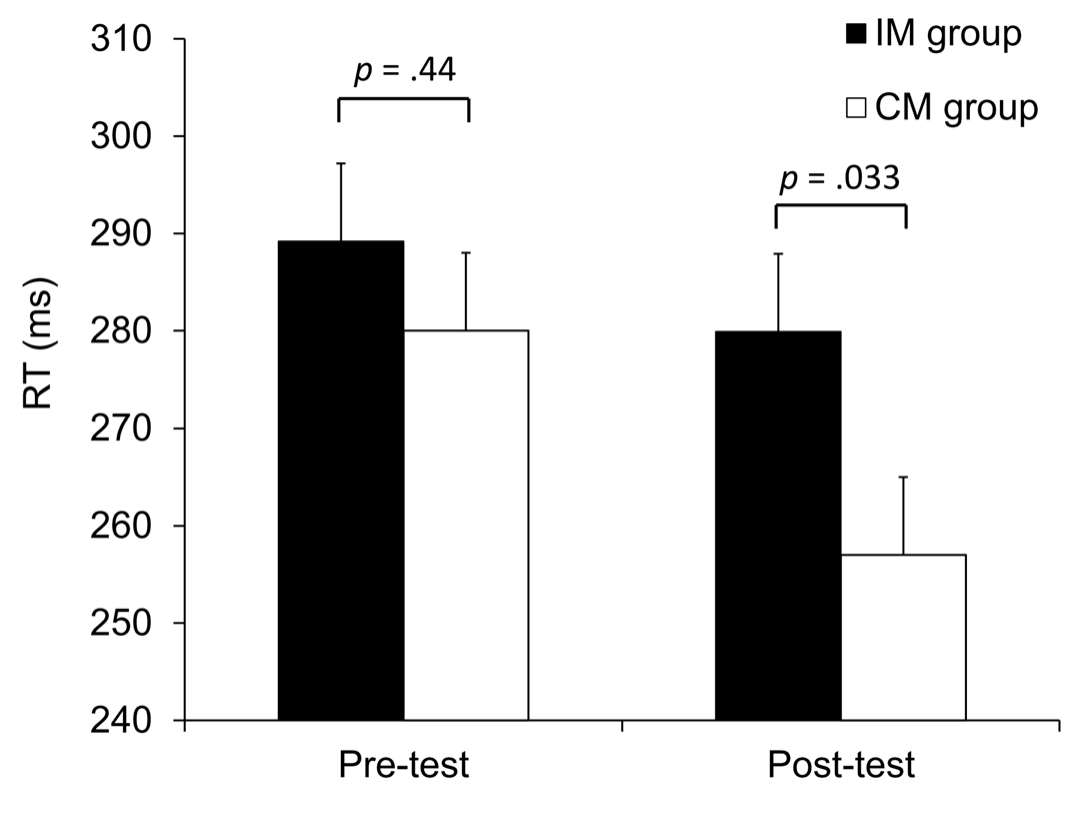
Pre- and Post-test mean RT in Experiment 1, for the Compatible Mapping (CM) group and Incompatible Mapping (IM) group. Vertical bars indicate the standard error of the mean. *p* values from the compatible vs. incompatible comparisons are given.

In order to test the influence of action-effect acquisition on intentional imitation performance, while controlling for potential differences in pre-test performance, we used the analysis of covariance approach [48–49]. Post-test RTs were entered into a univariate ANCOVA with Action-effect mapping (CM vs. IM) as the between-participants factor and pre-test mean RTs as the covariate. This analysis revealed a significant effect of Action-effect mapping, F(1, 37) = 4.88, *p* = .033, η ²_p_ = .12. The participants who received compatible mapping performed better (shorter RTs) than participants who received incompatible mapping (Fig. 3). The same analysis conducted on errors revealed no significant effect of Action-effect mapping, F(1, 37) = .54, *p* = .47, η ²_p_ = .01. Importantly, the pattern of errors was similar to that of the RTs, indicating no evidence of a speed—accuracy trade-off as an explanation of the observed RT effects.

Confirming our predictions, we found that intentional imitation was affected by the acquisition of action-effect associations through ideomotor learning. Imitative performance was better after compatible than incompatible-action-effect learning. This result can be interpreted as evidence for action—effect (or ideomotor) learning. For the CM group, the observed action in the imitation task was previously the effect of the required response. In contrast, for the IM group, the to-be-imitated action was previously the effect of a response which differs from the one required. Within the ideomotor framework, it is assumed that a learned action-effect activates the associated response. Thus, when performing the imitation task after compatible-action-effect learning, the perceived action primed the required response, facilitating imitation, whereas after incompatible acquisition, the observed action primed a different—incompatible—response, interfering with imitation. In line with earlier findings that action-effects can acquire the capacity to represent actions [12, 19–23], our results suggest that an observed movement processed as the effect of another action may later prime execution of that action.

Our imitation task involved viewing left and right hands. Consequently, when a right-hand was presented on the screen, there was spatial congruency between the observed movement and participant’s response, whereas when a left-hand was presented, there was spatial incongruency. Analysis of performance, across groups, pre- and post-tests, confirms that, compared to congruent trials, incongruent trials were indeed associated with longer RTs (306 vs. 248 ms), F(1, 39) = 285.99, *p* <. 001, η ²_p_ = .88, and higher error rates (7.8 vs. 1.1%), *F*(1, 39) = 134.30, *p* <. 001, η ²_p_ = .77.

Therefore, it is possible that the influence of learned action-effects on performance in the imitative task resulted from modulation of spatial congruency effects. Before acquisition, the congruency effect (difference between incongruent and congruent trials) for RT was similar in both groups (65 ms vs. 64 ms, for IM and CM groups, respectively), *F*(1, 38) = 0.04, *p* = .85, η ²_p_ = .00. The congruency effect in terms of error rates was also similar in both groups (6.2 vs. 8.3%, for IM and CM groups, respectively), *F*(1, 38) = 2.53, *p* = .12, η²_p_ = .06.

We performed an ANCOVA on the post-test congruency effects with Action-effect mapping (CM vs. IM) as a between-subjects factor and the congruency effect measured in pre-test as a covariate. This analysis revealed that the congruency effect measured in the CM group (49 ms) did not differ from that measured in the IM group (56 ms), *F*(1, 37) = 1.41, *p* = .24, η ²_p_ = .04. The same analysis on the congruency effects in terms of error rates revealed no difference between CM (6.6) and IM (5.5) groups, *F*(1, 37) = .96, *p* = .33, η²_p_ = .02.

Behavioral and neuroimaging studies have demonstrated that spatial congruency effects do not interact with imitative performance and are not mediated by the mirror-matching neural system [50–52]. The fact that we found no modulation of spatial compatibility by condition of action-effect acquisition suggests that our manipulation specifically affected processes responsible for the matching between executed and observed action.

## EXPERIMENT 2

Experiment 1 demonstrated that the ability to voluntarily imitate an observed movement can be affected by prior ideomotor learning. Experiment 2 aimed to reproduce and extend the results of Experiment 1 with a slightly different design. We modified the orientation of the hand stimuli in the imitation task in order to eliminate the spatial congruency effect. The observed hand on the screen was now displayed orthogonally to the participant’s hand (Fig. 1). This procedure was previously used by others to eliminate spatial congruency in imitation protocols [52].

Importantly, the orientation of stimuli in the acquisition phase remained unchanged, hence the hands observed during acquisition were oriented differently than those observed in the imitation task. This second experiment thus allowed us to test whether modulation of imitation by action-effect learning could be obtained even when there is not a strict visual correspondence between the stimuli observed during acquisition phase and imitation task. It also allowed us to further investigate the representations developed through ideomotor learning. An important aspect is indeed that only right hands were presented during the acquisition phase. If ideomotor learning creates perceptuomotor codes representing both the finger movement and the laterality of the observed hand, then subsequent imitation of right-hand finger movements should be specifically affected.

Finally, we also slightly reduced the amount of trials in both intentional imitation task and acquisition phase, since participants in Experiment 1 had informally described the experiment to be tiring and reported discomfort in their fingers.

### Method

#### Participants

Sixty-eight undergraduates (18 males), aged between 17 and 28 years (mean = 19.3 years) participated in the experiment, in exchange for course credit. All had normal or corrected-to-normal vision and were naïve with respect to the purpose of the experiment. Each participant read and signed an informed consent form prior to taking part in the experiment. Participants were randomly assigned to CM and IM groups.

#### Apparatus and Material

The apparatus and material (except orientation of hand stimuli in the imitation task) were the same as in Experiment 1.

#### Intentional imitation task

The task and procedures were identical to that used in Experiment 1, with the exception that the orientation of the observed hand was orthogonal to the participant’s hand (Fig. 1). Participants completed 4 blocks of 40 trials before and after action-effect acquisition. By excluding spatial compatibility from our design we hoped to reduce performance variability, which allowed us to reduce the amount of trials.

#### Acquisition phase

The procedure was the same as in Experiment 1 except that participants performed 9 blocks of acquisition trials and only right hands were presented as stimuli.

### Results and discussion

Three participants in Experiment 2 were excluded from all following analyses because they exhibited abnormal patterns during the acquisition phase: two participants showed more than 90% repeating responses and another participant showed 76% alternating responses. This exclusion affects none of the main results described below.

#### Acquisition

The participants produced, on average, 55% alternating responses. The repetition/alternation ratio was similar in IM and CM groups, Pearson's chi-square = 0.01, *p* = .94.

The mean RTs of the participants in the CM and IM groups were 380 ms (SD = 100) and 415 ms (SD = 111), respectively, but were not significantly different, *F*(1, 63) = 1.73, *p* = .19, η²_p_ = .027. An RT distribution analysis similar to that performed in Experiment 1 was conducted. Again, the difference between IM and CM groups tended to numerically increase with RT (IM mean RT—CM mean RT = 15 ms at bin 1, 21 ms at bin 2, 28 ms at bin 3, 40 ms at bin 4 and 58 ms at bin 5). However, the ANOVA, with Action-effect mapping (CM vs. IM) as between-participants factor and Bins (1–5) as within-participant factor, revealed a significant effect of Bins, *F*(4, 252) = 297, *p* <. 001, η²_p_ = .82, but no significant effect of Action-effect mapping, *F*(1, 63) = 1.73, *p* = .19, η²_p_ = .027, and no significant interaction between these two factors, *F*(4, 252) = 1.18, *p* = .32, η²_p_ = .018.

In both Experiment 1 and Experiment 2, the patterns of data in the acquisition phase were consistent with ideomotor predictions and previous demonstrations of action-effect compatibility [43–45, 53], but the statistical analyses did not reveal significant effects. However, it is important to note that during this phase, the participants were instructed to freely select a movement upon the arrival of an imperative stimulus, but they were not specifically instructed to initiate their response as fast as possible, which differs from the previous studies showing response-effect compatibility [43–45, 53]. The fact that response speed was not crucial in the acquisition phase makes RT a less reliable measure and may explain why RT analyses failed to reveal significant effects.

#### Intentional imitation task

Error trials (4.0%) were excluded from RT analyses. The same outlier procedure as in Experiment 1 was applied to the RT data, resulting in the exclusion of 2.57% of trials.

As in Experiment 1, we first analyzed pre-test data. An ANOVA conducted on mean RTs in pre-test with Action-effect mapping (CM vs. IM) as between-participants factor indicated no significant difference between groups, *F*(1, 63) = .38; *p* = .54, η²_p_ = .01. The same analysis on error rates revealed no significant difference between IM (3.5%) and CM (4.5%) groups, *F*(1, 63) = 2.46, *p* = .12, η²_p_ = .04.

**Fig 4:**
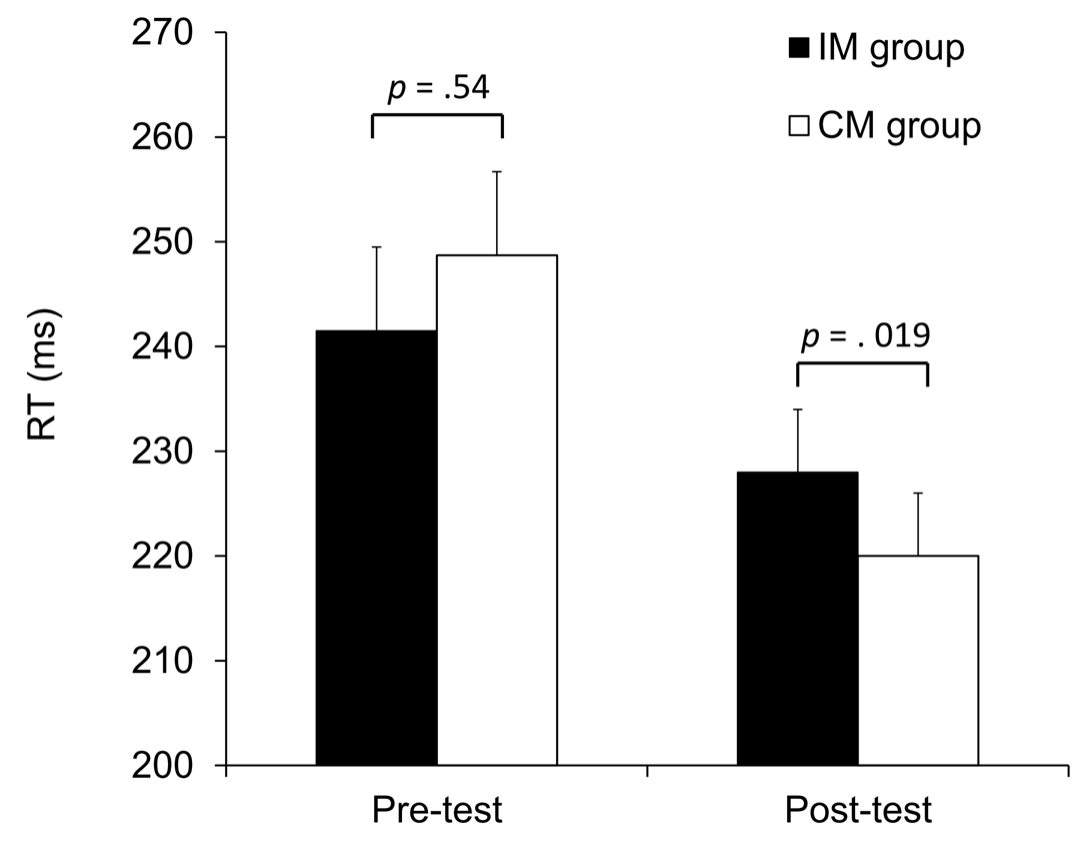
Pre- and Post-test mean RT in Experiment 2, for the Compatible Mapping (CM) group and Incompatible Mapping (IM) group. Vertical bars indicate the standard error of the mean. *p* values from the compatible vs. incompatible comparisons are given.

In order to evaluate the influence of action-effect acquisition on imitative performance, we performed an ANCOVA on the post-test RTs with Action-effect mapping (CM vs. IM) as a between-subjects factor and the pre-test RTs as a covariate. This analysis indicated a significant effect of Action-effect mapping, *F*(1, 62) = 5.79, *p* = .02, η²_p_ = .09. Participants in the CM group obtained better performance (shorter RTs) than participants in the IM group (Fig. 4). The same analysis on error rates (3.8% and 4.1% for IM and CM groups, respectively) revealed no effect of training, *F*(1, 62) = .55, *p* = .46, η²_p_ = .01. The error pattern confirms that the RT results cannot be attributed to a speed-accuracy trade-off.

This experiment replicates results from Experiment 1, showing that action-effect learning affects participants’ performance in an intentional imitation task. After a compatible-action-effect learning phase, where participants’ movements were paired with observation of matching movements, imitative performance was improved compared to performance following an incompatible learning phase where participants’ movements were paired with observation of non-matching movements.

In Experiment 2, the hands observed during the acquisition phase were oriented differently from those observed in the imitation task. In addition, only right hands were presented during the acquisition phase while both right- and left-hand finger movements had to be subsequently imitated. Imitation performance was nonetheless affected by prior action-effect acquisition.

A complementary analysis of the influence of Action-effect mapping on post-test RTs was conducted with the inclusion of Laterality of the observed hand (left vs. right) as a within-subjects factor. This analysis revealed that participants were slower to imitate left- than right-hand finger movements, 232 ms vs. 217 ms, respectively, *F*(1,62) = 11.84, *p* = .001, η²_p_ = .16. There was a significant effect of Action-effect mapping, *F*(1,62) = 5.88, *p* = .02, η²_p_ = .09, which was not modulated by Laterality of the observed hand, *F*(1,62) = .2, p = .61, η²_p_ = .01. Imitative capabilities of right- and left-hand finger movements were thus equally affected by action-effect learning which involved execution and perception of finger movements of the right hand only.

This result might indicate that action-effect learning created an integrated sensorimotor representation coding features of action such as finger identity, but not the laterality of the observed hand. Another possibility is that all features of actions and effects were integrated, with different weights being assigned to the represented features. This interpretation is consistent with previous findings and theoretical accounts suggesting that action-effect learning is mediated and contextualized through an ‘‘intentional-weighting” mechanism [33, 54–56]. This mechanism operates on the representations of the features of action and effects, giving more weight to those feature dimensions that are relevant for intended actions [54–56]. In our experiment, participants produced actions with their right-hand fingers only (in both the acquisition phase and the imitation task). In addition, the acquisition phase took place after a first test of imitation in which laterality of the observed hand was irrelevant (participants were to copy the finger movements, whenever a right or left hand was observed). Therefore, it is possible that during action-effect learning, the laterality dimension was given a small weight compared to finger identity. Finally, this finding is also consistent with neurophysiological studies of motor resonance—a correlate of the common coding between action and perception. It has been found that observation of right hand movements can trigger motor activity in both hemispheres [57] and left pre-motor cortex has been found to be activated both by observation of right and left hand movements [58], confirming that motor resonance does not necessarily encode the laterality of the observed hand.

## GENERAL DISCUSSION

The goal of this study was to test whether imitation could be modified by prior action-effect learning, as predicted by the ideomotor approach to action and perception. In two experiments, we contrasted the influence of two types of action-effect acquisition on subsequent intentional imitation performance. In the compatible mapping condition, the execution of an action (index or little finger lifting) was associated with the observation of a similar action. In the incompatible condition, the executed action (e.g. little finger lifting) was paired with observation of a different action (index finger lifting). In both experiments, in line with our predictions, we found that imitative performance was better after compatible-action-effect learning than after incompatible-action-effect learning. Our study demonstrates for the first time that such a type of action-effect learning can influence imitative behavior, providing a new line of evidence in support of the ideomotor account of imitation [2, 25].

The ideomotor theory suggests that imitation is made possible by action-effect associations, which are coded in integrated sensorimotor representations [2, 9, 33]. These representations are used for action control as well as action perception, solving the “correspondence problem”. It is further claimed that action-effect associations are built through experience. The idea is that agents are continuously integrating the perceptual codes of the consequences of their movements with the motor commands that brought them about [2, 11]. In our experiments, the acquisition phase with incompatible response-effect mapping is supposed to have established new associations between the motor codes of action “A” and the visual code of action “B”. Once established, this new link should interfere with imitation of B, since excitation of the visual representation of B induced excitation of the motor commands for execution of A. Thus, we expected a decreased imitative performance following this incompatible acquisition phase, compared to that measured after acquisition of compatible action-effect, which involved correlated experience of observing and executing the same action. The two experiments reported here confirmed this prediction.

The present work reveals that imitation can be modified by action-effect associations acquired by ideomotor learning. This finding is consistent with previous work on intentional imitation showing that neural processing of the to-be-imitated actions is influenced by prior sensorimotor experience with these actions [59–60].

This finding is also in agreement with current ideomotor theories, which emphasize the importance of action-effects in the control of action [2, 9]. In line with this, studies on ideomotor learning have demonstrated how remote action effects can acquire the capacity to prime the action that brought them about [12, 19, 21–23, 34–35]. In these studies, the executed action was typically a key press, which triggered an effect such as a sound [12] or a colored visual stimulus [61]. However, our work suggests that the ideomotor logic also applies to body-related effects of a movement, such as seeing the effector moving. Our results further suggest that this type of learned action effect may be particularly important for control and imitation of non-goal directed, intransitive actions.

By showing an influence of action-effect learning on the imitation of specific body movements, we also provide support for a recent model of imitative learning built on the ideomotor approach [38]. Accordingly, imitative learning is made possible by the acquisition of cascading bidirectional action—effect associations through observation of one’s own and others’ actions. The observation of one’s own movements leads to the acquisition of first-order action-effect associations, which link motor codes to the action’s typical visual effects, which includes the visual representation of the effector displacement. When perceiving another individual executing an action, the motor code corresponding to this action is activated in the observer, i.e. motor resonance, because of first-order associations. This motor code is then linked to the salient effects produced by the observed action in the environment. This creates second-order action-effect associations enabling later imitation of the observed action [25]. Experimental evidence for the acquisition of second order associations has been provided by recent work demonstrating the acquisition of action-effect association through observation of another’s action [31]. The present results may be seen as an illustration of the acquisition of first-order action-effect associations: we demonstrated ideomotor learning which linked an executed action with its visual effects consisting in effector displacements.

Furthermore, our results confirm and extend those from a recent study by Wiggett et al. [37] which showed an effect of sensorimotor experience on automatic imitation using an ideomotor learning procedure similar to that used in the present work. In this study, the authors found that after a learning phase where hand movement triggered observation of foot movement, the automatic imitation induced by observation of hand movements was reduced. In our study, participants in the incompatible mapping group showed impaired performance on the intentional imitation task compared to the compatible mapping group. Thus, combined together, our results and those reported by Wiggett et al. [37] support the hypothesis that common motor resonance processes are at the core of both intentional and automatic imitation [1, 13, 39–40]. Furthermore, separate studies on automatic and intentional imitation have found that these imitative behaviors were altered by disruption of the functioning of IFG (a part of the putative mirror system) with repetitive transcranial magnetic stimulation [42, 62–63]. Nevertheless, direct evidence that intentional and automatic imitations are both supported by similar mirror matching processes is still lacking. Only few studies have contrasted the brain activity associated with intentional vs. automatic imitation, and they have yielded conflicting results, with studies showing common and others distinct cerebral substrates [42, 64]. A future line of research to clarify this issue could test the influence of action-effect learning on both types of imitation in the same study.

The common representational basis for action execution and action perception implied by the ideomotor theory echoes with the mirror matching system theory that has been fuelled by the discovery of mirror neurons [6, 13–14]. The mirror neuron system (MNS) is in fact considered a neural substrate of the functional principles postulated by ideomotor theories [13]. In line with this, one view is that the MNS subserves imitation in humans [6, 13]. In this perspective, our results may indicate that the functioning of the MNS can be modified by ideomotor learning. This view is consistent with studies suggesting that activity in the human MNS is modified by relatively brief periods of sensorimotor experience [65–68].

Finally, previous studies found that when a response is consistently followed by an incompatible effect, response initiation is slower than when it is followed by a compatible effect [43–44]. For example, in a study where participants responded to centrally presented stimuli, it was demonstrated that right key presses were initiated faster if they were followed by the appearance of objects located on the right than if they triggered effects on the left, and vice versa [44]. This kind of result supports a central claim of ideomotor theory, in which the representations of effects are associated with the response that brought them about and these representations are then automatically activated in the course of initiating this response [44]. In the present study, we found a non-significant tendency for longer RTs in the IM group compared to the CM group during the acquisition phase. This tendency is consistent with the idea that action-effect associations developed during ideomotor learning and affected the capability to initiate movements, as predicted by ideomotor theory. This brings about the question of whether the difference in imitative performance between the IM and CM groups was related to a modification of imitative capabilities or merely an alteration of the capability to initiate the to-be executed movement. Unfortunately, the present data do not allow us to answer directly this question, since we had no control condition testing for movement initiation. Future investigations could examine this issue by testing whether movements produced in response to other movements (imitative action) or to abstract stimuli (non-imitative action) are initiated more or less slowly following a learning phase where execution of these movements was associated with perception of different vs. similar body movements.

To conclude, our study demonstrates that action-effect learning can influence intentional imitation. In line with the ideomotor approach, this result suggests that imitation is supported by acquired excitatory links between the action’s motor codes and the codes of sensory action effects.
